# Hepatocyte-specific Cas9-mediated editing of G6pc and Slc37a4 elicits comparable biochemical and regulatory responses between glycogen storage disease (GSD) type Ia and Ib mice

**DOI:** 10.1016/j.molmet.2026.102393

**Published:** 2026-06-10

**Authors:** Kishore A. Krishnamurthy, Ruiqi Xiao, Martijn G.S. Rutten, Trijnie Bos, Aycha Bleeker, Mingjia Zhang, Hilda I. de Vries, Mirjam Koster, Nicolette Huijkman, Marieke Smit, Niels Kloosterhuis, Theo Boer, Bauke Schomakers, Michel van Weeghel, Bart van de Sluis, Justina C. Wolters, Barbara M. Bakker, Maaike H. Oosterveer

**Affiliations:** 1 Laboratory of Pediatrics, University of Groningen, University Medical Center Groningen, the Netherlands; 2 Laboratory of Cellular Senescence and Age-related Pathologies, European Research Institute for the Biology of Ageing, University Medical Center Groningen, the Netherlands; 3 Department of Laboratory Medicine, University of Groningen, University Medical Center Groningen, the Netherlands; 4 Laboratory Genetic Metabolic Diseases, UMC Amsterdam, the Netherlands; 5 Core Facility Metabolomics, UMC Amsterdam, the Netherlands; 6 United for Metabolic Diseases (UMD), the Netherlands; 7 Therapy Accelerator for Rare Diseases, Radboud University Medical Center, Nijmegen, the Netherlands; 8 School of Pure and Applied Sciences, RV University, Mysore Campus, 571302, India

**Keywords:** Hepatic GSD Ia vs GSD Ib, Sugar phosphates, *de novo* fatty acid biosynthesis, Inflammation, Liver disease, Transcriptomics, Proteomics

## Abstract

**Background/Objective:**

Glycogen storage disease type I (GSD I) is an autosomal recessive inborn error of carbohydrate metabolism. Patients with GSD type Ia and Ib exhibit overlapping and distinct symptoms and complications. Notably, GSD Ia patients show more severe hypertriglyceridemia and higher risk of hepatic tumors than GSD Ib patients.

**Methods:**

Given the liver's pivotal role in these processes, this study utilized hepatocyte-specific CRISPR/Cas9-mediated somatic gene editing to explore the pathophysiological and biochemical adaptations in hepatic GSD Ia and Ib side-by-side. Additionally, hepatic histology, transcriptomics, and proteomics analysis was performed.

**Results:**

Compared to controls, hepatic GSD Ia and Ib mice showed hepatomegaly, fasting hypoglycemia, hyperlactatemia, and increased uric acid in plasma, which was somewhat more pronounced in GSD Ia than Ib. Both GSD I subtypes showed similar reductions in hepatic acetyl-CoA precursor pool enrichment and increases in *de novo* biosynthesis of hepatic stearate and oleate. Interestingly, only GSD Ia mice showed mildly elevated plasma triglyceride and hepatic phosphate sugars. Metabolic changes were reflected at the transcriptomic and proteomic levels, with largely similar responses between GSD Ia and Ib livers. Moreover, altered mRNAs and protein levels related to nucleotide-binding oligomerization domain (NOD) signaling pathways, infection and inflammation, liver disease, and chemical carcinogenesis were somewhat more pronounced in hepatic GSD Ia than in GSD Ib mice.

**Conclusions:**

Overall, the metabolic disturbance was more severe in hepatocyte-specific GSD Ia than in GSD Ib mice, consistent with the clinical phenotype in patients. The metabolic disorders and specific metabolites, genes, and proteins identified in this study provided new insights into the pathophysiological and biochemical phenotypes of GSD I subtypes in the liver.

## Highlights

• In-depth screening of hepatocyte-specific GSD Ia and Ib mice for biochemical, physiological, and molecular responses.
• The developed GSD Ia and GSD Ib mice provide valuable models for further investigation in the molecular disease mechanisms.
• The identified differences provide leads for subtype specific susceptibility to longterm complications.

## 1 Introduction

Glycogen storage disease (GSD) type I is a rare, autosomal recessive, inborn error of carbohydrate metabolism with a prevalence of 1 in 100,000 newborns [1]. Biallelic variants of the gene encoding glucose 6-phosphatase-α (*G6PC1*) cause GSD type Ia (GSD Ia, OMIM: #232200) [1,2], while biallelic variants of the gene encoding the glucose 6-phosphate transporter (*SLC37A4*) lead to GSD type Ib (GSD Ib, OMIM: #232220) [1,3]. *G6PC1* is predominantly expressed in hepatocytes, kidney cells, and enterocytes of the upper duodenum, whereas *SLC37A4* is ubiquitously expressed [1,2]. The catalytic conversion of G6P to glucose requires the concerted action of *SLC37A4* and *G6PC1*, thereby enabling the final biochemical reactions of gluconeogenesis and glycogenolysis [1,2].

Individuals with GSD Ia and Ib show partially overlapping symptoms and complications, likely because G6PC1 and SLC37A4 act in tandem in hepatocytes, enterocytes, and kidney cells, and their activities in these cell types are mutually dependent [2,[4], [5], [6], [7]]. Common disease characteristics of GSD Ia and Ib are fasting hypoglycemia, hepatomegaly, hyperlipidemia, hyperuricemia, hyperlactatemia, nephromegaly, liver tumor formation, and growth retardation [6,7]. Interestingly, in clinical practice, GSD Ia patients more frequently show severe hypertriglyceridemia than GSD Ib patients, and hepatocellular adenoma (HCA) formation appears most prevalent in GSD Ia [2,8]. Preclinical studies in conditional knockout mice indicate that hypertriglyceridemia and liver tumor formation are caused by loss of G6PC functionality in hepatocytes [9]. Moreover, alterations in multiple intrahepatic signaling pathways, such as Carbohydrate Response Element Binding Protein (ChREBP)-dependent control of intrahepatic glucose and lipid metabolism and 5′AMP-activated Protein Kinase (AMPK), mechanistic Target of Rapamycin (mTORC1), and Sirtuin1 (SIRT1)-dependent regulation of hepatic autophagy, have been proposed to contribute to symptoms and complications in hepatic GSD Ia and Ib [[10], [11], [12], [13], [14], [15], [16], [17], [18], [19]]. Yet, the exact mechanisms underlying differences in hepatic symptoms and complications between GSD Ia and GSD Ib patients remain largely unresolved. Specifically, the biochemical and regulatory adaptations to hepatic GSD Ia and Ib have not been directly compared.

This study aimed to expand current insights into the pathophysiological adaptations in hepatic GSD Ia and GSD Ib. For this purpose, we performed side-by-side analysis of the biochemical, physiological, and molecular responses (metabolomics, *de novo* lipogenesis, untargeted transcriptomics and proteomics) to hepatocyte-specific somatic *G6pc* and *Slc37a4* editing in mice.

## 2 Materials and methods

### 2.1 Animal experimentation and sample collection

The animal study was approved under license #AVD10500202115288 by the Institutional Animal Care and the committee of the University of Groningen. Hepatocyte-specific Alb-Cas9-positive [20] male mice aged 8–10 weeks were used. Animals were divided into three groups of 10 mice each and injected intra-orbitally with serotype 8 adeno-associated viruses (AAV8; 1+E11 particles) under isoflurane anesthesia. The AAV8 viruses were produced in HEK293T cells and purified by iodixanol ultracentrifugation, as previously described [20,21]. The virus administered to the first experimental group contained 3 single guide RNAs (sgRNAs) targeting exon 1 of murine *G6pc* to generate hepatocyte-specific GSD Ia mice [22], that of the second group contained 3 sgRNAs targeting exon 3 of murine *Slc37a4* (sequences are listed in Table S1) to generate hepatocyte-specific GSD Ib mice*,* while the third group received an empty AAV8 expressing an empty U6-cassette [20] and served as the control group. This approach allowed for CRISPR/Cas9-mediated somatic *in vivo* gene editing of *G6pc* and *Slc37a4*. Specifically, this approach can be used to silence genes by the generation of DNA double-strand breaks in genes of interest, which (in the absence of a repair template) will be repaired by non-homologous end-joining (NHEJ), resulting in insertions and deletions (indels) that generally cause loss-of-function [23]. The experiment was performed in two separate animal cohorts that were subjected to the same experimental procedures with a 7-week interval in between. All animals were housed in individually ventilated cages (IVC) containing wood bedding, nesting wooden curls, and cardboard rolls in a light and temperature-controlled facility (12h light; from 8.00 AM to 8.00 PM/12h dark; from 8.00 PM to 8:00 AM, cycles) with *ad libitum* access to a regular chow diet (RM1 special diet service, UK) and drinking water available, unless stated otherwise. Blood glucose levels were measured in a drop of tail blood weekly during the first three weeks after AAV8 administration following a daytime fast (9 AM-3 PM, with access to drinking water) using a handheld glucometer (Roche, Mannheim, Germany). During the final 48 h before sacrifice, animals received 2% sodium 1–^13^C acetate (Sigma #13291-89-9; Merck Life Science, Amsterdam, The Netherlands) via their drinking water, allowing to quantify hepatic fatty acid synthesis rates [24,25]. Four weeks after virus administration, mice were overnight fasted (10 PM-9 AM, with access to drinking water), after which blood glucose levels were measured in a drop of tail blood. Animals were subsequently sacrificed by cardiac puncture under isoflurane anesthesia. Blood was collected in EDTA-coated tubes (#3072121 SARSTEDT AG & co. Numbrecht, Germany) and immediately stored on ice until centrifugation for 10 min at 4000×*g* at 4 °C, after which blood plasma was collected and stored at −80 °C until further analysis. Livers were dissected and partly fixed in formaldehyde (4% w/v in PBS). The remaining liver tissue was immediately freeze-clamped and temporarily stored on dry ice, after which samples were stored at −80 °C until further analysis.

### 2.2 Histological analysis of the liver

Fixed liver tissue was embedded in paraffin and sectioned (4 μm) using a microtome. The sections were stained with Hematoxylin and Eosin [26], and photographs were taken at 20x magnifications using a DFC420 Camera (Leica, Wetzlar, Germany).

### 2.3 Biochemical analysis

Hepatic G6P and glycogen levels were quantified in liver tissue using enzymatic methods as described [[27], [28], [29]]. Lipids were extracted from liver tissue, according to Bligh & Dyer, as described previously [30]. Hepatic triglyceride, total cholesterol, and free cholesterol [31] (Roche, Mannheim & Diasys, Holzheim) levels were subsequently quantified using commercially available kits. Hepatic cholesterol ester contents were calculated by subtracting free cholesterol from total cholesterol levels. Hepatic phospholipid contents were quantified as described [32]. These liver data were normalized to liver weight. Plasma lactate (#2897 Instruchemie, Delfzijl, The Netherlands), uric acid (#KA1651, Abnova, Taipei, Taiwan), and non-esterified fatty acid levels (#157819910935, Diasys, Holzheim, Germany) were analyzed using commercially available kits. Plasma triglyceride (Roche, Precimat glycerol standard (Roche) and cholesterol levels (Roche, cholesterol standard FS (DiaSys) were quantified according to the manufacturer's instructions.

### 2.4 Hepatic glucose 6-phosphatase activity

Microsomal phosphohydrolase activities in liver tissue were analyzed using a previously described procedure [9,33,34], in which microsomal membranes are permeabilized with sodium deoxycholate prior to activity assessment. Data were normalized to protein contents determined using the BCA assay kit (#A53225, Thermo Fischer, Rockford, USA). In microsomes isolated from the hepatic GSD Ib mice, there was a more than two-fold increase of the measured G6Pase activity upon addition of deoxycholate, consistent with a transport-dependent activity. The remaining activity in the absence of deoxycholate (Fig. S1) suggests some leakiness of the microsomes, probably due to freezing and thawing of the liver samples [35]. Since the assay was meant to measure the catalytic activity, not the transport activity, we report the total activity in the presence of deoxycholate, without further correction.

### 2.5 Hepatic gene expression analysis

RNA was extracted from liver tissue using TRI reagent (#T9424 Sigma–Aldrich) and quantified using Nanodrop (Thermo Fischer, Rockford, USA). 1000 ng of the extracted RNA was used to prepare complementary DNA (cDNA) using M-MLV (Invitrogen) per manufacturer's instructions. qPCR was performed to amplify the cDNA for targeted gene expression analysis using SYBR Green reagent (Table S2). Dilutions of a pool composed of all samples were used to prepare a standard curve for quantification of relative mRNA expression levels. Calculated relative mRNA levels were subsequently normalized to beta-actin expression and expressed relative to the average values of control mice. For untargeted transcriptome analysis, the quality of the extracted RNA was confirmed by gel electrophoresis (2% agarose gel) to ensure that the RNA was intact and suitable for sequencing. RNA library preparation and sequencing were carried out by Novogene Co. Ltd. Europe. Sequencing data were aligned and analyzed using the mouse reference genome (GRCm38) genome and gene model annotation files were downloaded from the genome and corresponding gene annotations obtained from public genome databases (NCBI/UCSC/Ensembl). The resulting raw gene count data were processed using Network Analyst 3.0 [36]. To improve data robustness, only genes detected in at least two-thirds of all samples were retained for downstream analysis. Missing values were handled using a stepwise approach. When possible, missing values were replaced with the average value of the corresponding experimental group. If no group-level data were available, missing values were replaced with one-fifth of the smallest detected value, which approximates the detection limit of the instrument. Gene expression data were then normalized, and differences between experimental groups were quantified using the EdgeR package in R (version 4.2.2) [37]. Expression changes were reported as log2 Fold Changes (Log2^FC^), and differentially expressed genes (DEGs) were assessed using false discovery rate (FDR) correction to account for multiple testing. Genes with an FDR ≤0.05 were considered differentially expressed. To interpret the biological relevance of the observed gene expression changes, pathway enrichment analysis was performed using the KEGG database via DAVID Bioinformatics Resources (version 2024q2). Finally, the results were visualized using standard graphical approaches, including principal component analysis (PCA), volcano plots and bar plots generated with commonly used R packages "ggplot2" [38], as well as heatmap by "pheatmap" [39].

### 2.6 Western blot analysis

Liver tissue was lysed using RIPA buffer (50 mM Tris–HCl (pH 7.4), 150 mM NaCl, 1% Nonidet P-40, 1 mM PMSF, 2 mM EDTA, 50 mM sodium fluoride (NaF), 0.2 mM sodium orthovanadate (Na_3_VO_4_), and 1X Complete protease inhibitor cocktail (Roche Diagnostics, Mannheim, Germany), and homogenized using a bead beater (at 6000 Hz, 2x for 15 s each). The homogenate was incubated on ice for 30 min, with vortexing every 10 min, and subsequently centrifuged at a maximum speed (13,523×*g*) at 4 °C for 10 min. The supernatant was collected and used for protein quantification using the Lowry assay (#5000111 Biorad). The lysate was subsequently diluted to 25 μg protein/10 μL using RIPA and Laemmli loading buffer (#161–0747 Biorad with 10% beta-mercaptoethanol, #60-24-2, Biorad). The samples were boiled at 95 °C for 5 min, loaded onto acrylamide gels (with the gel percentages tailored to the target proteins), and subjected to electrophoresis at 70 V for the first 20 min, followed by 100 V for 150 min. Separated proteins were then transferred onto PVDF membranes (#A29574727, Amersham™ Hybond™^,^ GE Healthcare Life Science) by tank blotting for 120 min at 45 V, with ice packs placed in the transfer tank. Membranes were blocked using either 5% BSA (#90604-29-8, Carl ROTH® Karlsruhe Germany) in Tris-Buffered Saline with 1% tween (1% TBST) or 5% milk powder (Campina, Netherlands) in 1% TBST for 1 h at room temperature under agitation. The membranes were incubated overnight at 4 °C with specific primary antibodies (Table S3). After incubation, the membranes were washed 3 times for 10 min at room temperature. The membranes were then incubated with suitable secondary antibodies (Table S3) for 90 min at room temperature. Again, membranes were washed 3 times for 10 min with 1% TBST at room temperature. Protein bands were detected after incubation with Enhanced Chemi Luminescence (ECL) reagent (#A38554, Pierce Biotechnology, Rockford, IL, USA) using an ImageQuant imager, GE LAS 4000 mini machine (Cytiva, GE Healthcare, Uppsala, Sweden). Protein band intensities were quantified along with the rubber band method of background subtraction using Imagequant software (Version 8.2) (GE Healthcare Europe GmbH; Freiburg, Germany). For data analysis, a previously described approach was used [40]. Pixel intensities of each signal were first normalized to the mean pixel intensity of all signals corresponding to the respective readout within a given experiment. These values were subsequently normalized to the loading control (i.e., HSP90 or β-actin). Finally, relative phospho- and total protein levels were calculated from these double-normalized values.

### 2.7 Untargeted proteomics analysis

Liver homogenates were 10% (w/v%) prepared as described above for western blot analysis. Total protein concentration was quantified using Pierce™ BCA Protein Assay Kit (Thermo Scientific). To prepare the samples for mass spectrometry, homogenates were delipidated and proteins were digested into peptides following established protocols [41]. The resulting peptide mixtures were collected, dried, and resuspended in 0.1% v/v formic acid at a final concentration of 25 ng/μL, calculated based on the protein concentration before delipidation. Aliquots of 20 μL peptide solutions were loaded onto Evotips (Evosep Aps, Odense C, Denmark) for chromatographic separation. Peptides were separated using Evosep One HPLC (Evosep Aps, Odense C, Denmark) equipped with a C18 column. Mass spectrometry analysis was performed on the Orbitrap Exploris 480 (Thermo Scientific) coupled with a FAIMS Pro interface (Thermo Scientific), which was used to improve ion selection and signal quality. Data were acquired using a 44-minute HRMS1-DIA method. The raw data obtained from mass spectrometry were processed in Spectronaut v.16 (SN16) using a library-free approach (directDIA). Protein identification was based on curated mouse protein sequences from the UniProt database (17021 entries). For the downstream analysis and data visualization, proteins were annotated with their corresponding gene names, and quantitative values derived from integrated peak areas by SN16 were further processed in RStudio, using the same analytical pipeline as described above for transcriptomics.

### 2.8 Targeted proteomics analysis

Quantitative protein levels of G6PC and SLC37A4 were determined using targeted proteomics on protein digests from the untargeted proteomics samples as described previously [41]. Isotopically-labeled standard peptides GLGVDLLWTLEK (100 fmol ^13^C^15^N-lysine labeled Pepotec-Grade 2, Thermo Scientific) for G6PC and AGLSLYGNPR (10 fmol ^13^C^15^N-arginine labeled Pepotec-Grade 2, Thermo Scientific) for SLC37A4 were measured together with 1 μg total protein digest for the quantification of these two proteins.

### 2.9 Quantification of various total hepatic fatty acids synthesis rates

Hepatic lipids were hydrolyzed and derivatized as described [42]. The fatty acid mass isotopomer distributions were determined by gas chromatography-mass spectrometry (GCMS) and used in mass isotopomer distribution analysis to calculate acetyl-CoA precursor pool enrichments, *de novo* fractional fatty acid synthesis rates, as well as the fractions of oleate and stearate generated by chain elongation of pre-existing palmitate [42]. Hepatic contents of individual fatty acids were quantified by gas chromatography after transmethylation [43]. Absolute fatty acid synthesis was calculated by multiplying fractional synthesis rates of specific fatty acids with the corresponding hepatic fatty acid contents.

### 2.10 Hepatic metabolomics analysis

Semi-targeted metabolomics analysis of frozen liver samples was performed using liquid chromatography coupled to mass spectrometry (LC-MS) with internal standards as described [44]. Corrected metabolite data were expressed relative to the average values of control mice.

### 2.11 Statistical analysis

Graphs present data from individual mice from both animal cohorts, and mean values of each experimental group are indicated with their respective standard errors of the mean. Differences between groups were analyzed by one-way ANOVA followed by Tukey post-hoc pairwise comparisons, using a significance level of *p* < 0.05. The statistical analysis applied on transcriptomics and proteomics were performed using R (v4.2.2), with specific packages applied as described in the respective sections [[37], [38], [39]].

## 3 Results

### 3.1 CRISPR/Cas9-mediated gene editing successfully induced hepatic GSD Ia and GSD Ib, with GSD Ia mice showing larger livers and temporarily more severe fasting hypoglycemia than GSD Ib mice

**Figure 1:**
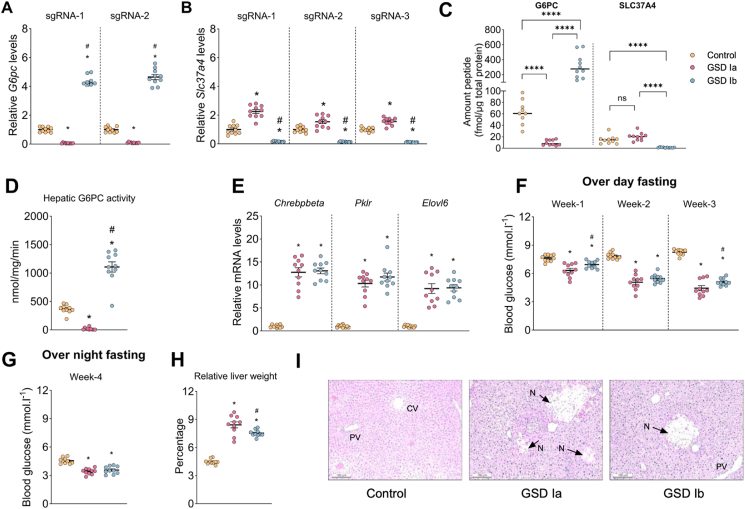
Relative hepatic mRNA levels of (**A**) G6pc and **B**) Slc37a4, using primer sets amplifying the regions targeted by the respective sgRNAs. (**C)** G6PC and SLC37A4 protein levels as determined by targeted proteomics. (**D)** Maximal phosphohydrolase activity in microsomal extracts from liver tissue. (**E)** Relative hepatic mRNA levels of Chrebp-beta, Pklr, and Elovl6. (**F)** Blood glucose levels at 1, 2, and 3 weeks after sgRNA administration, measured after a daytime fast (9AM-3PM). (**G)** Blood glucose levels at 4 weeks after sgRNA administration, measured after an overnight fast (10PM-9AM). (**H)** Liver-to-body weight ratios, expressed as percentages. **(I)** Representative pictures of Hematoxylin and Eosin (H&E) stainings on liver tissue sections showing the necrotic patches (N), central vein (CV) and portal vein (PV) with a scale bar of 100 μM. Data from individual mice are plotted, and the mean values of each experimental group are indicated. Orange circles mark data from control mice; pink circles mark data from GSD Ia mice; blue circles mark data from GSD Ib mice. Differences between groups were analyzed by one-way ANOVA, post-hoc Tukey's multiple comparison test, using a significance level of p < 0.05. ∗ marks significant differences between hepatic GSD Ia or GSD Ib and control mice. # marks significant differences between hepatic GSD Ia and GSD Ib mice.

In order to evaluate and compare the biochemical, physiological, and molecular responses to hepatocyte-specific *G6pc* and *Slc37a4* deletion *in vivo*, we employed somatic CRISPR/Cas9-mediated gene editing in mice. Quantitative PCR analysis confirmed that treatment of hepatocyte-specific Cas9-expressing mice with sgRNAs directed against murine *G6pc* (for GSD Ia) or murine *Slc37a4* (for GSD Ib) resulted in strong reductions in hepatic mRNA levels of the regions targeted by the respective sgRNAs (Figure 1A,B). In addition, the hepatic *G6pc* mRNA level was induced in hepatic GSD Ib mice, and analogously the *Slc37a4* mRNA level was induced in hepatic GSD Ia mice (Figure 1A,B). Quantification of hepatic G6PC and SLC37A4 protein by mass spectrometry analysis showed clear reduction of G6PC and SLC37A4 levels in the respective GSD I mouse models compared to the controls (Figure 1C). Hepatic G6PC activity in GSD Ia mice was strongly and significantly reduced compared to controls, resulting in residual activities between 19 % and 1% (Figure 1D). Both G6PC protein level and enzymatic activity were markedly elevated in hepatic GSD Ib mice relative to controls (Figure 1C,D). It should be noted that, as microsomal membranes were permeabilized prior to G6PC activity assessment [9,33,34], the observed increase in hepatic G6PC activity in GSD Ib mice likely reflects an artefact related to this assay condition. In line with previous studies [12,22,45,46], the mRNA levels of the glucose-sensitive transcription factor *Chrebpβ,* as well as that of its target genes *Pklr* and *Elovl6*, were strongly and similarly increased in both GSD Ia and Ib mice (Figure 1E).

During the first three weeks after sgRNA administration, blood glucose levels decreased significantly and progressively in overday-fasted hepatic GSD Ia and GSD Ib mice compared to controls (Figure 1F). After one and three weeks, hepatic GSD Ib mice showed slightly higher blood glucose levels than hepatic GSD Ia mice (Figure 1F). After four weeks, overnight fasted blood glucose levels were significantly reduced in hepatic GSD Ia and Ib mice compared to controls, with no difference observed between GSD Ia and Ib mice (Figure 1G).

After four weeks, the liver-to-body weight ratios were significantly higher in GSD Ia and Ib mice than in controls, while they were also significantly higher in hepatic GSD Ia than in GSD Ib mice (Figure 1H). Histological analysis of liver tissue sections revealed cytoplasmic vacuolation in hepatocytes and signs of necrotic foci in GSD Ia and Ib livers (Figure 1I). In contrast, liver sections from control mice showed normal liver architecture and the absence of cytoplasmic vacuolation (Figure 1I).

Altogether, these findings indicate that CRISPR/Cas9-mediated *G6pc* and *Slc37a4* somatic editing in livers induced hypoglycemia, hepatomegaly, and hepatocellular vacuolation in mice, symptoms corresponding with hepatic GSD Ia and GSD Ib in humans. Furthermore, hepatic GSD Ia mice showed slightly more severe hypoglycemia at specific timepoints, and somewhat more pronounced hepatomegaly at sacrifice than GSD Ib mice.

### 3.2 Differential elevation of hepatic phosphate sugars distinguishes GSD Ia from Ib in mice with comparable glycogen and lactate accumulation

**Figure 2:**
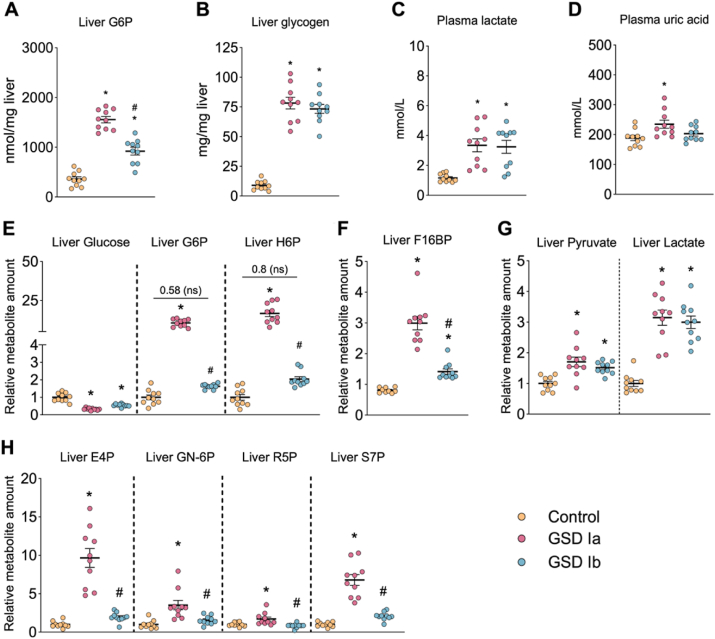
Hepatic (**A)** Glucose 6-phosphate (G6P) and (**B)** glycogen contents. Plasma (**C)** lactate and (**D)** uric acid concentrations. Hepatic levels of (**E)** glucose, G6P, and hexose 6-phosphate (H6P), (**F)** fructose 1,6-bisphosphate (F16BP), (**G)** pyruvate and lactate, (**H)** erythrose 4-phosphate (E4P), gluconate 6-phosphate (GN-6P), ribose 5-phosphate (R5P), and sedoheptulose 7- phosphate (S7P). Data presented in A-D were generated using biochemical assays and data presented in E-H are determined by Mass Spectrometry analysis. Data from individual mice are plotted, and the mean values of each experimental group are indicated. Orange circles mark data from control mice; pink circles mark data from GSD Ia mice; blue circles mark data from GSD Ib mice. Liver data were normalized to liver weight. Differences between groups were analyzed by one-way ANOVA, post-hoc Tukey's multiple comparison test, using a significance level of p < 0.05. ∗ marks significant differences between hepatic GSD Ia or GSD Ib and control mice. # marks significant differences between hepatic GSD Ia and GSD Ib mice.

We next evaluated liver and plasma metabolite concentrations in hepatic GSD Ia and GSD Ib. Enzymatic analysis showed a significant increase in hepatic G6P content in both GSD Ia and Ib mice compared to controls, with significantly higher G6P levels in hepatic GSD Ia than GSD Ib mice (Figure 2A). The glycogen content in GSD Ia and Ib mouse livers increased equally compared to the controls (Figure 2B). Plasma lactate levels were significantly and similarly increased in hepatic GSD Ia and Ib mice (Figure 2C), while plasma uric acid levels were increased in hepatic GSD Ia mice only (Figure 2D). Semi-targeted LC-MS analysis revealed significantly reduced intrahepatic free glucose levels in GSD Ia and Ib mice compared to controls (Figure 2E), mirroring the blood glucose levels (Figure 1G). In agreement with the enzymatic analysis (Figure 2A), the metabolomics analysis showed a slightly more pronounced increase of hepatic G6P and hexose 6-phosphate levels in GSD Ia mice compared to GSD Ib mice, which was just not significant in GSD Ib (p = 0.58 and p = 0.8, respectively) (Figure 2E). Fructose 1,6-bisphosphate (F1,6BP) levels were increased in both GSD Ia and Ib mice, with GSD Ia mice showing significantly higher F1,6BP levels than GSD Ib mice (Figure 2F). Hepatic pyruvate and lactate levels were significantly increased in both hepatic GSD Ia and GSD Ib mice compared to controls (Figure 2G).

In parallel, hepatic levels of the pentose phosphate pathway (PPP) intermediates erythrose 4-phosphate (E4P), gluconate 6-phosphate (GN-6P), ribose 5-phosphate (R5P), and sedoheptulose 7-phosphate (S7P) were significantly increased in hepatic GSD Ia compared to controls (Figure 2H). A similar, though not significant, trend was observed in GSD Ib mice for E4P, GN-6P, and S7P levels.

In addition, *H6pd*, the gene encoding ER-localized hexose-6-phosphate dehydrogenase, was found up-regulated in both GSD Ia and Ib (log2^FC^ = 0.62 in GSD Ia vs Control and log2^FC^ = 0.69 in GSD Ib vs Control), with a slightly more pronounced increase observed in GSD Ib than in GSD Ia (log2^FC^ = −0.08 in GSD Ia vs Ib) (Table S5). Although the corresponding H6PD protein levels do not reach statistical significance, a consistent increase is observed (log2^FC^ = 0.18 in GSD Ia vs Control and log2^FC^ = 0.13 in GSD Ib vs Control), with a slightly more pronounced increase observed in GSD Ia (log2^FC^ = 0.05 in GSD Ia vs Ib) (Table S6).

These data show that, although hepatic glycogen and lactate similarly accumulated in hepatic GSD Ia and Ib mice compared to the controls, the increase of phosphorylated intermediates of glycolysis, PPP, and the *H6pd* gene expression in liver, as well as that of plasma uric acid, was somewhat more pronounced in GSD Ia than GSD Ib.

### 3.3 Hepatic GSD Ia and Ib mice showed similar increases in hepatic *de novo* lipogenesis and medium-chain acylcarnitine contents, while plasma triglyceride levels were significantly increased in GSD Ia mice only

**Figure 3:**
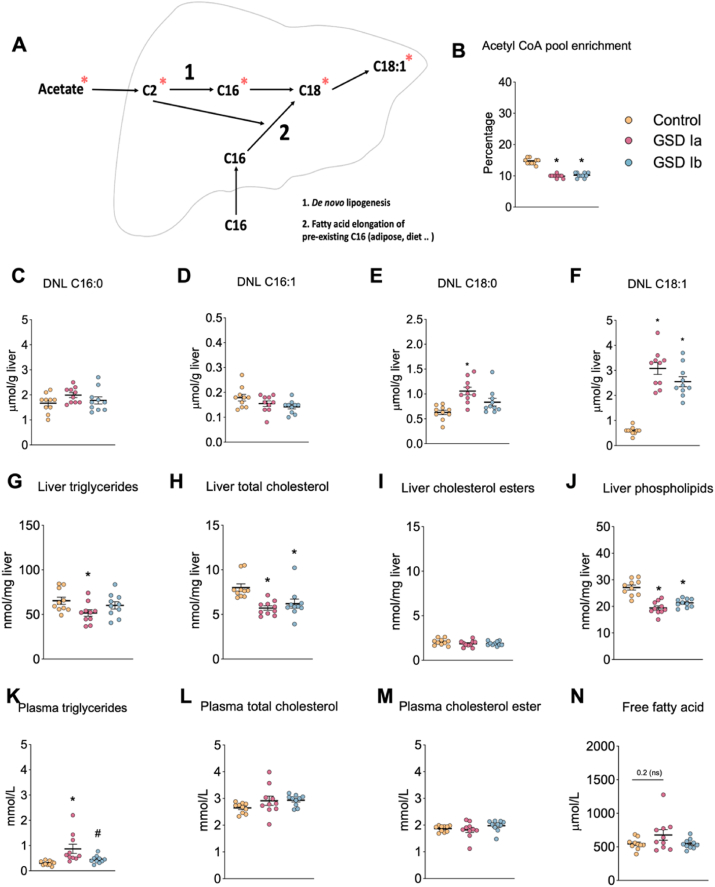
(**A**) Schematic representation of the approach used to estimate hepatic *de novo* lipogenesis upon 1–^13^C acetate incorporation into hepatic fatty acids. (**B**) Acetyl-CoA precursor pool enrichment in hepatic palmitate expressed as a percentage. (**C**) Absolute *de novo* lipogenesis (DNL) of hepatic palmitate (C16:0), (**D**) palmitolate (C16:1), (**E**) stearate (C18:0), and (**F**) oleate (C18:1). Hepatic concentrations of (**G**) triglyceride, (**H**) total cholesterol, (**I**) cholesteryl-ester, and (**J**) phospholipid contents. Plasma (**K**) triglyceride, (**L**) total cholesterol, (**M**) Cholesteryl-ester, and (**N**) free fatty acid concentrations. Data in panels C–F represent absolute fatty acid synthesis after 48 h of sodium 1-[^13^C] exposure. Data from individual mice are plotted, and the mean values of each experimental group are indicated. Orange circles mark data from control mice; pink circles mark data from GSD Ia mice; blue circles mark data from GSD Ib mice. Liver data were normalized to liver weight. Differences between groups were analyzed by one-way ANOVA, post-hoc Tukey's multiple comparison test, using a significance level of p < 0.05. ∗ marks significant differences between hepatic GSD Ia or GSD Ib and control mice. # marks significant differences between hepatic GSD Ia and GSD Ib mice.

We then evaluated hepatic lipid metabolism parameters that relate to hepatic steatosis and hypertriglyceridemia, two hallmarks of hepatic GSD Ia and GSD Ib [9]. In order to quantify hepatic fatty acid synthesis from *de novo* lipogenesis and elongation of pre-existing fatty acids, animals received ^13^C-labeled acetate via their drinking water [42], after which lipids were hydrolyzed and ^13^C label incorporation in specific fatty acids was determined using mass spectrometry analysis (Figure 3A).

**Figure 4:**
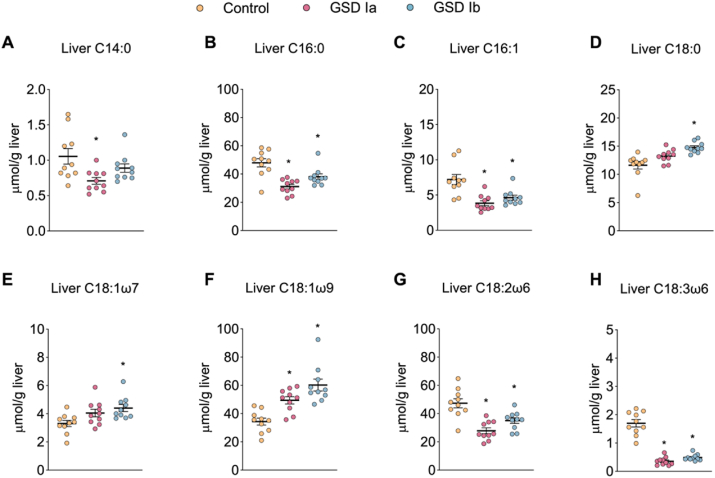
**Levels of the total amount of each specific hepatic fatty acid after hydrolysis of the hepatic lipids, determined by Gas Chromatography.** Hepatic contents of **(A)** myristate (C14:0), **(B)** palmitate (C16:0), **(C)** palmitoleate (C16:1), **(D)** stearate (C18:0), **(E)** vaccenate (C18:1ω7), **(F)** oleate (C18:1ω9), **(G)** linoleate (C18:2ω6), and **(H)** gamma-linolenate (C18:3ω6) as determined by Gas Chromatography analysis. Data from individual mice are plotted, and the mean values of each experimental group are indicated. Orange circles mark data from control mice; pink circles mark data from GSD Ia mice; blue circles mark data from GSD Ib mice. Liver data were normalized to liver weight. Differences between groups were analyzed by one-way ANOVA, post-hoc Tukey's multiple comparison test, using a significance level of p < 0.05. ∗ marks significant differences between hepatic GSD Ia or GSD Ib and control mice. # marks significant differences between hepatic GSD Ia and GSD Ib mice.

Based on the isotope incorporation in hepatic palmitate, it was estimated that hepatic acetyl-CoA pool enrichments were significantly reduced in both GSD Ia and Ib mice compared to controls (Figure 3B). *De novo* lipogenesis (DNL) of hepatic palmitate (C16:0) and palmitoleate (C16:1) remained unchanged in hepatic GSD Ia and GSD Ib mice (Figure 3C,D), while *de novo* stearate (C18:0) synthesis was significantly increased in GSD Ia only (Figure 3E), and oleate/vaccenate (C18:1 ω7/ω9) synthesis from DNL was significantly higher in both GSD Ia and Ib mice than controls (Figure 3F). The fractional and absolute contributions also showed elevated chain elongation of the pre-existing fatty acids in both GSD Ia and Ib mice compared to controls (Table S4). In contrast to earlier findings [22], hepatic triglyceride contents, were mildly but significantly reduced in hepatic GSD Ia mice but not in GSD Ib mice compared to the controls (Figure 3G). Furthermore, hepatic cholesterol and phospholipid contents were significantly reduced in both GSD Ia and Ib mice compared to the control mice, while cholesterol ester contents remained unaffected (Figure 3H-J). In parallel, plasma triglyceride levels were significantly increased in GSD Ia but not in GSD Ib mice compared to controls (Figure 3K). Plasma total cholesterol, cholesterol ester, and free fatty acid levels were similar between groups (Figure 3L-3N). The hepatic C14:0 contents were significantly reduced in GSD Ia mice only (Figure 4A), while C16:0 and C16:1 contents were significantly reduced in both GSD Ia and Ib mice livers (Figure 4B,C). On the contrary, hepatic C18:0, C18:1ω7, and C18:1ω9 contents were increased in both GSD Ia and Ib mice (Figure 4D–F), corresponding to the elevated *de novo* lipogenesis of C18:0 and C18:1 (Figure 3E,F). Interestingly, hepatic C18:2ω6 and C18:3ω6 contents were significantly reduced in GSD Ia and Ib mice as compared to controls (Figure 4G,H).

**Figure 5:**
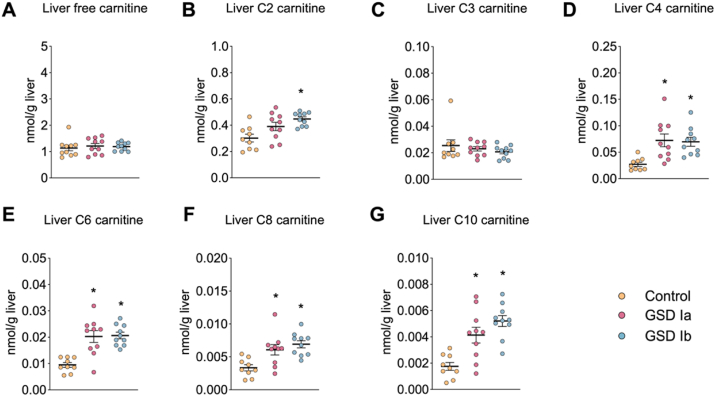
**Levels of various hepatic carnitines determined by Mass Spectrometry.** Hepatic levels of (**A)** free carnitine, (**B)** C2-carnitine, (**C)** C3-carnitine, (**D)** C4-carnitine, (**E)** C6-carnitine, (**F)** C8-carnitine, and (**G)** C10-carnitine as determined by Mass Spectrometry analysis. Data from individual mice are plotted, and the mean values of each experimental group are indicated. Orange circles mark data from control mice; pink circles mark data from GSD Ia mice; blue circles mark data from GSD Ib mice. Liver data were normalized to liver weight. Differences between groups were analyzed by one-way ANOVA, post-hoc Tukey's multiple comparison test, using a significance level of p < 0.05. ∗ marks significant differences between hepatic GSD Ia or GSD Ib and control mice. # marks significant differences between hepatic GSD Ia and GSD Ib mice.

Acylcarnitines are the carriers of fatty acids and translocate long-chain fatty acids to the mitochondria for the β-oxidation. Accumulation of acylcarnitines may suggest impaired fatty acid oxidation [47]. Hepatic free and C3-carnitine levels were similar between the groups, while C2-carnitine levels were modestly increased in hepatic GSD Ia mice and significantly increased in GSD Ib mice compared to controls (Figure 5A-C). Hepatic C4-, C6-, C8-, and C10-carnitine levels were significantly increased in both hepatic GSD Ia and Ib mice compared to controls (Figure 5C–G), partly in line with the previous literature [12].

In summary, hepatic fatty acid synthesis was increased while acylcarnitines, the intermediates of fatty-acid oxidation, accumulated in hepatic GSD Ia and Ib mice compared to the controls. Notably, hepatic GSD Ia mice demonstrated significantly increased levels of plasma triglycerides, rendering their biochemical phenotype more severe than that of GSD Ib mice.

### 3.4 Hepatic GSD Ia and GSD Ib mice show similar reductions in hepatic pAMPK/AMPK and pULK1/ULK1 ratios and elevated hepatic p62 protein levels

**Figure 6:**
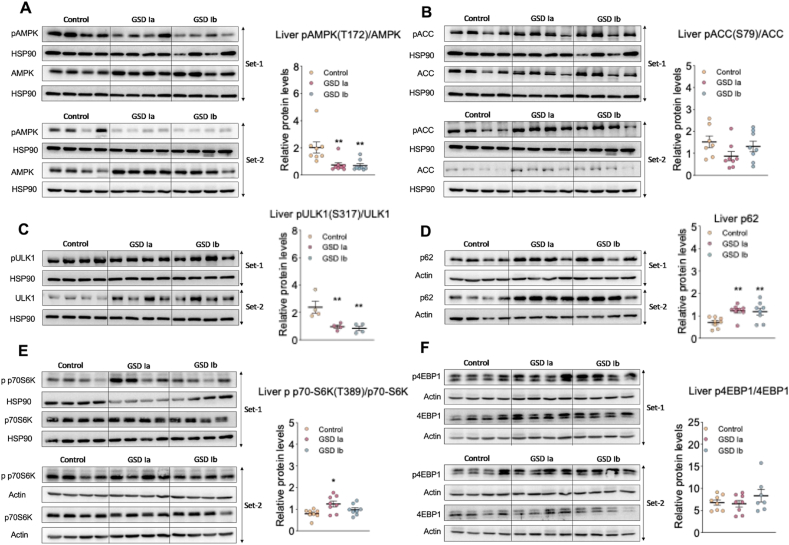
**Hepatic GSD Ia and GSD Ib mice show similar reductions in hepatic pAMPK/AMPK and pULK1/ULK1 ratios and elevated hepatic p62 protein levels**. Western blot images depicting hepatic (**A**) AMPK, p-AMPK (T172) and HSP90 protein levels (left) and p-AMPK/AMPK protein ratios (right), (**B)** ACC, p-ACC (Ser79) and HSP90 protein levels (left) and p-ACC/ACC protein ratios (right), (**C)** 4EBP1, p-4EBP1 (Thr37/46) and beta-actin protein levels (left) and p-4EBP1/4 EBP1 protein ratios (right), (**D)** p70S6K, p-p70 S6K (T389), HSP90 and b-actin protein levels (left) and p-p70S6K/p70S6K protein ratios (right), (**E**) ULK1, p-ULK1 (S-317) and HSP90 protein levels (left) and p-ULK1/ULK1 protein ratios (right), and **(F)** p62 and Actin protein levels (left) and p62/actin protein ratios (right). For each protein Western Blot analysis, a total of 8 samples per group were run on 2 independent polyacrylamide gels, generating 2 sample sets (referred to as Set-1 and Set-2). Intensity of the phosphorylated proteins were normalized to the loading control (either HSP90 or actin) on that membrane, and intensity of the corresponding total protein was also normalized to the loading control (either HSP90 or actin) on that membrane – normalized values are used to calculate the phospho-to-total ratio. Notably, phosphorylated and total protein were run on different gels. Similarly, the graphs present data from individual mice, and the mean values of each experimental group are indicated. Orange circles mark data from control mice; pink circles mark data from GSD Ia mice; blue circles mark data from GSD Ib mice. Liver data were normalized to liver weight. Differences between groups were analyzed by one-way ANOVA, post-hoc Tukey's multiple comparison test, using a significance level of p < 0.05. ∗ marks significant differences between hepatic GSD Ia or GSD Ib and control mice.

Autophagy has been previously reported to be compromised in hepatic mouse models for GSD Ia and Ib [[15], [16], [17], [18]]. We therefore compared autophagy-regulating signaling pathways in the livers of hepatic GSD Ia and GSD Ib mice. Western blot analysis showed that pAMPK/AMPK ratios were significantly reduced in both hepatic GSD Ia and Ib mice liver as compared to controls (Figure 6A). The pULK1(S317)/ULK1 ratio, a regulatory node downstream of pAMPK, which induces autophagy, was significantly reduced in both hepatic GSD Ia and Ib mice compared to controls (Figure 6C). Hepatic pACC/ACC ratios, another downstream target of pAMPK that reduces *de novo* lipogenesis, were non-significantly reduced in hepatic GSD Ia mice compared to the controls (Figure 6B). In line with the reduced ULK1 phosphorylation, hepatic protein levels of p62, were significantly increased in both GSD Ia and Ib mice compared to controls (Figure 6D). Furthermore, compared to controls, the hepatic p-p70S6K/p70S6K ratios, a downstream target of mTORC1 signaling, was slightly, but significantly increased in hepatic GSD Ia mice but not in hepatic GSD Ib mice (Figure 6E). In contrast, hepatic p4EBP1/4EBP1 levels, another downstream target of mTORC1, remained unaffected in hepatic GSD Ia and Ib mice compared to controls (Figure 6F).

Altogether, these signaling data suggest that upstream activators of hepatic autophagy is are equally impaired in hepatic GSD Ia and Ib mice compared to the controls, which is most likely related to downregulated AMPK phosphorylation, with a possible contribution of mTORC1 activation in GSD Ia mice.

### 3.5 Transcriptomics and proteomics analysis identifies highly overlapping pathway alterations in hepatic GSD Ia and GSD Ib mice

In order to obtain a more profound insight into the regulatory and signaling pathways contributing to symptoms and complications in hepatic GSD Ia and Ib, we performed transcriptomics and proteomics of livers of hepatic GSD Ia, GSD Ib, and control mice. Although experiments were conducted using two separate animal cohorts, heatmap and Principal Component Analysis (PCA) analysis of all detected 17962 genes and 3242 proteins revealed no obvious batch effects (Fig. S2). Therefore, the two batches were combined for downstream analysis.

**Figure 7:**
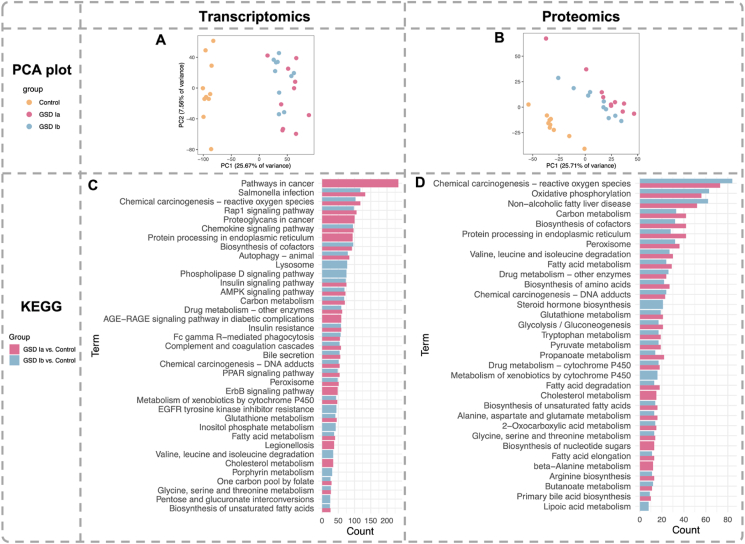
Overview of transcriptomics and proteomics results. (**A** and **B**) Principal component analysis (PCA) plot of three conditions in genes and proteins. **(C** and **D)** KEGG pathway enrichment analysis of significantly changed genes and proteins in transcriptomics and proteomics. Too generic pathways (‘metabolic pathways’ in contrast to a specific metabolic pathway) or too specific, but off-topic pathways (neurodegeneration diseases, osteoclast differentiation, fluid shear stress and atherosclerosis, and retinol metabolism) were excluded from the figures.

The PCA visualization shows a clear separation between GSD I and controls, in both hepatic genes (Figure 7A) and proteins (Figure 7B), but only in proteomics data GSD Ia was clearly distinct from GSD Ib. Interestingly, the two subtypes separated in the same direction as the separation between control and GSD I, in agreement with our observations above that the hepatic GSD Ia mice showed a somewhat more severe phenotype than their GSD Ib counterparts. Compared to control mice, hepatic GSD Ia mice exhibited 7602 differentially expressed genes (DEGs) (Fig. S3A) and 1157 significantly changed proteins (SCPs) (Fig. S3B). In hepatic GSD Ib mice, 694 DEGs (Fig. S3C) and 815 SCPs (Fig. S3D) were identified compared to controls. Direct comparison between GSD Ia and GSD Ib mice revealed 79 DEGs (Fig. S3E) and 26 SCPs (Fig. S3F). For transcriptome and proteome, log2^FC^, p-values, and FDR values of different comparisons are summarized in Sheets 1–3 of Tables S5 and S6, respectively.

To first explore the pathophysiological mechanisms of GSD I, irrespective of the subtype, we analysed which KEGG pathways were differentially regulated in GSD Ia relative to control, and Ib relative to control (Sheets 1 and 2 in Tables S7 and S8). Out of the top 30 FDR-ranked KEGG pathways, 23 pathways (77%) were shared between GSD Ia and Ib relative to control in transcriptomics (Figure 7C) and 27 (90%) pathways in proteomics (Figure 7D). Pathways related to the metabolism of glucose, propanoate, pyruvate, cholesterol, and fatty acids were significantly changed in both GSD Ia and Ib, in agreement with previous studies [[1], [2], [3], [4], [5], [6]] and with the reported metabolomic data in this study. Also, autophagy- and AMPK-related genes were changed in both GSD Ia and Ib. In untargeted proteomics, however, autophagy was not identified as an altered pathway, while the AMPK signaling pathway was only significantly changed in GSD Ib proteomics compared to control (FDR = 0.02), but not in GSD Ia versus control (FDR = 0.12) (Table S8). Other pathways related to liver function, such as chemical carcinogenesis, reactive oxygen species and drug metabolism, were changed in both transcriptomics and proteomics in both GSD I types compared to controls, and oxidative phosphorylation and non-alcoholic fatty liver disease only in proteomics.

In conclusion, integrated KEGG pathway analysis of transcriptomics and proteomics data reveals a high degree of similarity in metabolic process changes in hepatic GSD Ia and Ib mice, which is in line with the observed metabolic changes.

### 3.6 Genes and proteins related to NOD signaling pathway, infection and inflammation, liver disease, and chemical carcinogenesis were changed in hepatic GSD Ia versus GSD Ib mice

**Figure 8:**
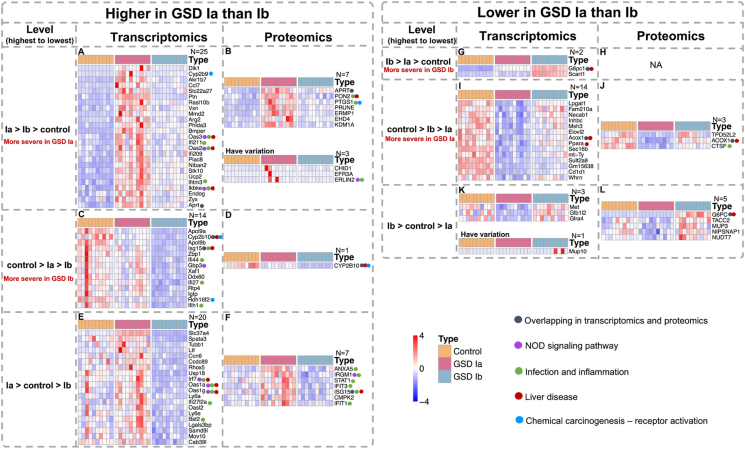
Heatmap of significantly changed genes and proteins (FDR <0.05) between hepatic GSD Ia and Ib livers. ‘N’ represents the number of genes or proteins involved. 'Ia > Ib > control' refers to the category in which the genes/proteins levels were highest in GSD Ia, lower in Ib, and lowest in the control group.

To further explore the metabolic profiles of hepatic GSD Ia and GSD Ib, we identified 79 DEGs and 26 SCPs between these two subtypes, with 5 of them overlapping (Figure 8). Initially, these DEGs and were classified based on whether their detected intensity levels were higher or lower in GSD Ia than in GSD Ib. Subsequently, they were further grouped according to the regulation pattern across control, GSD Ia, and GSD Ib conditions.

The largest number of genes and proteins were found in the category in which the levels were highest in GSD Ia, lower in Ib and lowest in the control group (Ia > Ib > control) (Figure 8A,B). In contrast, when levels were lower in GSD Ia than in GSD Ib, the largest subcategory was control > Ib > Ia (Figure 8I,J). In line with the previous, in most cases, GSD Ia and Ib mice showed the same direction in responses in gene and protein categories, with GSD Ia mice responding more strongly than GSD Ib mice. Apart from G6PC itself, four genes/proteins showed similar responses at the transcriptome and the proteome levels, i.e., APRT, CYP2B10, ISG15, and ACOX1 (labeled in grey in Figure 8). In our study, both the gene and protein levels of APRT (adenine phosphoribosyl transferase) were elevated in GSD Ia and Ib, with a slightly more pronounced increase in GSD Ia (Figure 8A,B). CYP2B10 and ACOX1, which are associated with lipid accumulation [48,49], showed reduced transcript and protein levels in both GSD Ia and Ib. In addition, *Isg1*5 mRNA levels were decreased in both subtypes (Figure 8C), whereas ISG15 protein levels showed divergent patterns, being elevated in GSD Ia and reduced in Ib (Figure 8F).

Given the very few DEGs and SCPs, the pathway annotation was manually curated based on differentially regulated KEGG pathways in the transcriptome (Sheet 3 in Table S7) and individual protein functions (Table S9). Among the genes and proteins that were higher expressed in GSD Ia than in GSD Ib, several were involved in the NOD (Nucleotide-binding Oligomerization Domain) signaling pathway (7 genes and 2 proteins), infection and inflammation (14 genes and 10 proteins), liver disease (11 genes and 5 proteins), and chemical carcinogenesis - receptor activation (2 genes and 2 proteins). Among the (fewer) genes that were higher in GSD Ib than in GSD Ia, the only common KEGG pathway was very generic (‘liver disease’).

In summary, relatively few differences were observed between GSD Ia and Ib at the transcriptome and proteome level, and these do not seem to explain the observed modest differences in sugar phosphate accumulation between GSD Ia and Ib livers. The genes and proteins that were altered between hepatic GSD Ia and Ib mice point to a specific activation of the NOD signaling pathway, infection and inflammation, and chemical carcinogenesis - receptor activation in GSD Ia. We note, however, that only few genes and proteins in these pathways were differentially regulated between the two GSD I subtypes. The more severe up- or down-regulation of genes and proteins in GSD Ia compared to Ib is consistent with our finding that GSD Ia mice suffer from slightly more pronounced liver symptoms.

## 4 Discussion

In this study, two somatic editing models for hepatocyte-specific *G6pc* and *Slc37a4* knockout mice were investigated to get insight into pathophysiological adaptations in GSD Ia and Ib mouse livers. The strengths of this study are (i) that hepatic GSD Ia and Ib models were phenotyped side-by-side within the same genetic background, and (ii) the comprehensive multi-omics characterisation. The phenotypes of the hepatic GSD Ia and GSD Ib models were largely comparable and characterized by reduced fasting blood glucose, elevated liver weight, hepatic glucose 6-phosphate (G6P) and reduced AMPK phosphorylation. Overall, the metabolic phenotype was somewhat more pronounced in hepatic GSD Ia compared to Ib mice, and the metabolic hallmarks were reflected in transcriptomics and proteomics, with larger changes in GSD Ia than GSD Ib livers compared to controls. Nevertheless, while the study demonstrates metabolic similarities between hepatic G6PC1 and SLC37A4 inactivation in mice, it does not provide clear explanations for the phenotypic differences observed between patients with GSD Ia and GSD Ib.

GSD Ia mice compensated hepatic *G6pc* editing by *Slc37a4* induction, while GSD Ib mice exhibited elevated G6PC mRNA and peptide levels. The latter resulted in elevated G6Pase activity in GSD Ib liver. The adapted gene expression may reflect a compensatory response to altered phosphate sugar levels. Elevated G6PC levels in GSD Ib mice can be explained by the fact that *G6pc* is a direct target gene of ChREBP [[50], [51], [52]], In line with this, we observed that the transcript levels of *Chrebpβ* and its target genes *Pklr* and *Elov6* were significantly increased in GSD Ib livers compared to controls [12,13]. In contrast *Slc37a4* is not transcriptionally regulated by ChREBP [10], but its concentration is regulated by elevated G6P [10].

Our finding that the metabolic, proteomic and transcriptomic phenotype of the somatic edited-hepatic GSD Ia mouse model is somewhat more pronounced than that of the GSD Ib mouse model is consistent with the more severe hepatic symptoms of GSD Ia versus GSD Ib patients [2,8]. Yet, comparative transcriptomics and proteomics did not explain the exacerbated overflow of G6P into the PPP and *de novo* lipogenesis in GSD Ia compared to Ib. The relatively small transcriptomic and proteomic changes may further suggest that direct enzyme kinetic responses in response to the higher G6P concentration in GSD Ia livers contributed substantially to the subtle metabolic differences between the subtypes. Possibly, *G6PC* exerts more control over hepatic G6P and its downstream pathways than *SLC37A4* [53]. Hepatic *G6pc* and *Slc37a4* editing almost completely abolished transcript levels of the targeted gene regions and resulted in average reductions of 84% and 92% in G6PC and SLC37A4 peptide levels, respectively. It should be noted that editing efficiency, mosaicism, and indel structure were not assessed at the DNA level. Because of these unquantified limitations, it should be emphasized that the subtle differences observed between hepatic GSD Ia and GSD Ib mice in our study may partly be related to residual function or mosaicism.

Finally, accumulated G6P may show different subcellular distributions in the two GSD I subtypes, with a shift towards the endoplasmic reticulum (ER) in GSD Ia compared to Ib. Although is unclear how this affects metabolic regulation, there is evidence that there is PPP activity within the ER [54,55], which may play a role particularly in GSD Ia. The elevated H6PD gene and protein levels observed in GSD Ia and Ib compared with controls, support the notion of up-regulated ER-PPP in these disease states.

Both GSD Ia and GSD Ib livers showed elevated p62 protein levels. In line with previous literature [[15], [16], [17], [18]], hepatic GSD Ia and Ib mice showed significantly reduced ratios of pAMPK/AMPK and its downstream target pULK1(S317)/ULK1 [56] compared to controls. The fact that these changes were similar in hepatic GSD Ia and Ib mice suggests that AMPK-ULK1 signaling responds to the same cues in hepatic GSD Ia and Ib mice. The role of mTORC1 in hepatic GSD Ia is more controversial. Some studies showed increased mTORC1 activity in GSD Ia [18], in agreement with elevated ratios of its target p70S6K/70S6K in our study. In contrast, others reported reduced mTORC1 activities [18] or marginal changes in GSD Ia [15]. The reason for the discrepancy warrants detailed analysis of metabolites and hormones, as mTORC1 activity is regulated by many different inputs [[56], [57], [58]]. Hepatic p70S6K was unaffected in GSD Ib, while p4EBP1, another downstream target of mTORC1, was unaffected in both GSD Ia and Ib mice. This further supports the notion that subtle and possibly conflicting metabolic inputs determine the mTORC1 activity in hepatic GSD I mice. Moreover, it is conceivable that the differential activation of p70S6K and 4EBP1 in our study reflects context-dependent regulation of these proteins by mTORC1. Finally, we observed that these pathways were significantly regulated at the mRNA level in both hepatic GSD Ia and GSD Ib mice relative to controls.

A characteristic feature of both GSD Ia and Ib that was replicated in the current study is hepatocyte vacuolation. [15,16,22]. An unexpected observation, though, was the presence of necrotic patches in hepatic GSD Ia and Ib mice, but not in controls. This was paralleled by increased expression of genes involved in TNF-a and NFkB signaling in hepatic GSD Ia and Ib mice. Previously, it has been reported that whole-body GSD Ia mouse livers show signs of necrosis and immune cell infiltration at 6 weeks after birth [59] and it has been speculated that this immune cell infiltration marks hepatocellular adenomas and carcinoma initiation [[59], [60], [61]], Furthermore, glycogen and lipid-mediated inflammation has been reported in GSD Ia [4,62]. In contrast, other liver-specific GSD Ia mouse models showed no signs of hepatic inflammation [12,22]. We previously used adenoviruses (AV) for CRISPR/Cas9-mediated *G6pc* editing [20], while here we have used adeno-associated viruses (AAVs) [63]. AAVs may modulate the immune microenvironment, leading to increased immune cell infiltration [64,65]. It is conceivable, therefore, that the observed necrosis is related to the combined effect of the AAV and *G6pc/Slc37a4* deficiency.

Another puzzling finding was that hepatic triglyceride levels were reduced in hepatic GSD Ia mice compared to controls. This contrasts our previous study, in which hepatic triglyceride levels were almost doubled in hepatic GSD Ia mice [22]. One potential explanation is that the AAVs used in the current study initiate gene editing from the second week after administration [66], while the adenoviral sgRNA vectors used previously [22] become functional within the first week of injection, potentially resulting in a more pronounced lipid phenotype.

The slightly more pronounced metabolic, proteomic and transcriptomic changes are in line with the more severe liver phenotype of GSD Ia compared to GSD Ib patients. The observed differences between the two mouse models are subtle, however. Pathways that have been implicated in tumorigenesis, such as elevated lactate production [22] were similarly affected in GSD Ia and Ib. The main differences were the slightly more pronounced increase of glycolytic and PPP metabolites, expression of a key PPP gene *H6pd*, *de novo* lipogenesis and plasma triglycerides in hepatic GSD Ia compared to Ib mice. Interestingly, compared to hepatic GSD Ib mice, mRNA and protein analysis marked increased NOD signaling, infection and inflammation, liver disease, and chemical carcinogenesis - receptor activation in hepatic GSD Ia mice. These pathways have a critical role in chronic liver diseases and hepatocellular carcinoma [[67], [68], [69]]. Thus, besides higher phosphorylated sugars in hepatic GSD Ia mice, these adaptations may sensitize GSD Ia mice to tumorigenesis, hence contributing to the higher risk of GSD Ia patients to develop liver adenomas and carcinomas [14,[70], [71], [72]].

APRT, CYP2B10, ISG15 and ACOX1, the shared DEGs and SCPs, indicated potential alterations in AMP biosynthesis, hepatic lipid metabolism, and innate immunity in GSD I. Although no studies have linked these genes/proteins to GSD I, their molecular functions allow us to speculate on their potential roles in GSD I pathophysiology. APRT regulates purine metabolism and AMP biosynthesis via a salvage pathway, which is energetically less costly than *de novo* AMP synthesis. [73]. The slightly more pronounced increase in APRT gene and protein levels in GSD Ia aligned with our previous finding of increased plasma uric acid in GSD Ia mice, suggesting that up-regulation of APRT may help recycle adenine into AMP, reducing its conversion into uric acid and supporting nucleotide and energy balance. CYP2B10 and ACOX1 are known to be decreased in fatty liver conditions, contributing to hepatic steatosis [48,49]. Therefore, reduced CYP2B10 and ACOX1 levels may have contributed to impaired hepatic lipid metabolism in hepatic GSD I mice. Interestingly, *Isg1*5 mRNA levels were decreased in hepatic GSD Ia and Ib, but the ISG15 protein level showed divergent patterns, suggesting post-transcriptional regulation. As ISG15 plays a central role in the innate immunity and inflammation [[74], [75], [76], [77]] the contrasting patterns of ISG15 protein expression in GSD Ia and Ib mice suggest distinct immune or metabolic adaptations between these two subtypes.

In summary, this study revealed that hepatocyte-specific GSD Ia and Ib mice somatic gene editing show largely comparable biochemical, physiological, and molecular responses, with GSD Ia mice showing somewhat more pronounced accumulation of phosphate sugars, increased lipogenesis, and increased NOD signaling, inflammation response, liver disease, and chemical carcinogenesis. We propose that these thoroughly and systematically characterized hepatic GSD I mouse models provide valuable models for further investigations into the molecular mechanisms underlying chronic complications and for identifying biomarkers to improve monitoring of GSD Ia and GSD Ib patients.

## Documentation of approval from the institutional committee for care and use of laboratory animals (or comparable committee)

All experimental procedures were approved by the Institutional Animal Care and Use Committee of the University of Groningen (Groningen, The Netherlands) under permit number AVD10500202115288, and are in line with the Guide for the Care and Use of Laboratory Animals.

## CRediT authorship contribution statement

**Kishore A. Krishnamurthy:** Writing – original draft, Visualization, Methodology, Investigation, Formal analysis, Data curation, Conceptualization. **Ruiqi Xiao:** Writing – original draft, Visualization, Methodology, Investigation, Formal analysis, Data curation, Conceptualization. **Martijn G.S. Rutten:** Investigation, Formal analysis, Data curation. **Trijnie Bos:** Investigation, Formal analysis. **Aycha Bleeker:** Investigation, Formal analysis. **Mingjia Zhang:** Visualization, Data curation. **Hilda I. de Vries:** Investigation. **Mirjam Koster:** Formal analysis. **Nicolette Huijkman:** Methodology, Investigation. **Marieke Smit:** Methodology, Investigation. **Niels Kloosterhuis:** Investigation. **Theo Boer:** Investigation, Formal analysis. **Bauke Schomakers:** Methodology. **Michel van Weeghel:** Methodology. **Bart van de Sluis:** Methodology. **Justina C. Wolters:** Writing – review & editing, Supervision. **Barbara M. Bakker:** Writing – review & editing, Supervision, Project administration, Funding acquisition, Conceptualization. **Maaike H. Oosterveer:** Writing – review & editing, Supervision, Project administration, Funding acquisition, Conceptualization.

## Ethics statement

The data were obtained by experiment and data analysis. All experimental procedures involving animals were approved by the Institutional Animal Care and Use Committee of the University of Groningen (Groningen, The Netherlands) under permit number AVD10500202115288, and are in line with the Guide for the Care and Use of Laboratory Animals.

## Data sharing statement

The mass spectrometry proteomics data have been deposited to the ProteomeXchange Consortium via the PRIDE partner repository with the dataset identifier PXD066807.

## Funding information

This study was supported by European Union's Horizon 2020 research and innovation program under the Marie Skłodowska-Curie grant agreement PoLiMeR #812616, Sophie's Hope Foundation, the De Cock-Hadders Foundation and ZonMw VIDI #91717373. Ruiqi Xiao and Mingjia Zhang were supported by the China Scholar Council (CSC) for their doctoral studies at University of Groningen (202106220094 and 202308230138 respectively).

## Declaration of competing interest

The authors declare that they have no known competing financial interests or personal relationships that could have appeared to influence the work reported in this paper.

## Data Availability

Processed data has been added in the supplementary tables. The raw proteomics data have been deposited to the ProteomeXchange Consortium via the PRIDE partner repository with the dataset identifier PXD066807.
