# Supplementary material for: Hepatocyte-specific Cas9-mediated editing of G6pc and Slc37a4 elicits comparable biochemical and regulatory responses between glycogen storage disease (GSD) type Ia and Ib mice
Source: Mol Metab. 2026 Jun 10;110:102393. doi: 10.1016/j.molmet.2026.102393 (PMC13316303; doi:10.1016/j.molmet.2026.102393)
Supplement: Supplement Figures [file mmc10.docx]

**
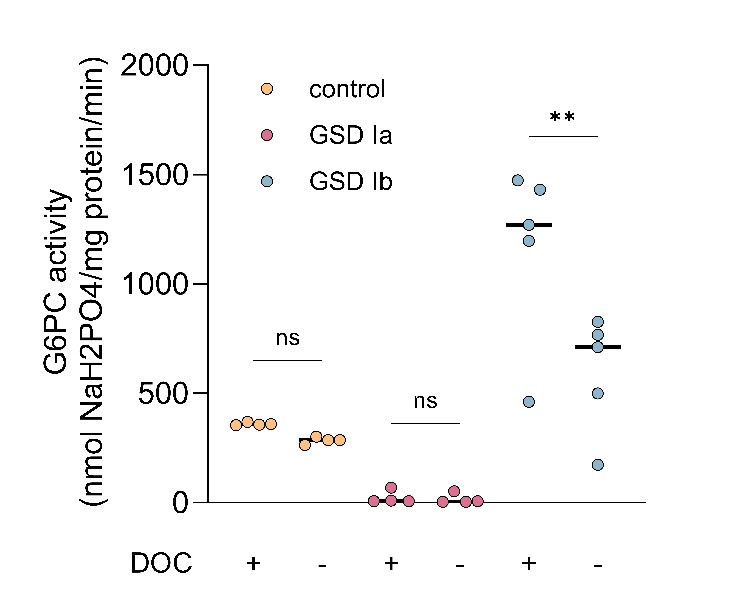
**

**Figure S1.** microsomal G6Pase activity in the absence and the presence of the detergent deoxycholate (DOC)**.**

Data from individual mice are plotted, and the mean values of each experimental group are indicated. Orange circles mark data from control mice; pink circles mark data from GSD Ia mice; blue circles mark data from GSD Ib mice. Differences between the presence and absence of deoxycholate in each group were analyzed by one-way ANOVA, post-hoc Tukey's multiple comparison test. A significance of ** marks p < 0.01 and “ns” marks no significant differences between the presence or absence of deoxycholate in this group.

**
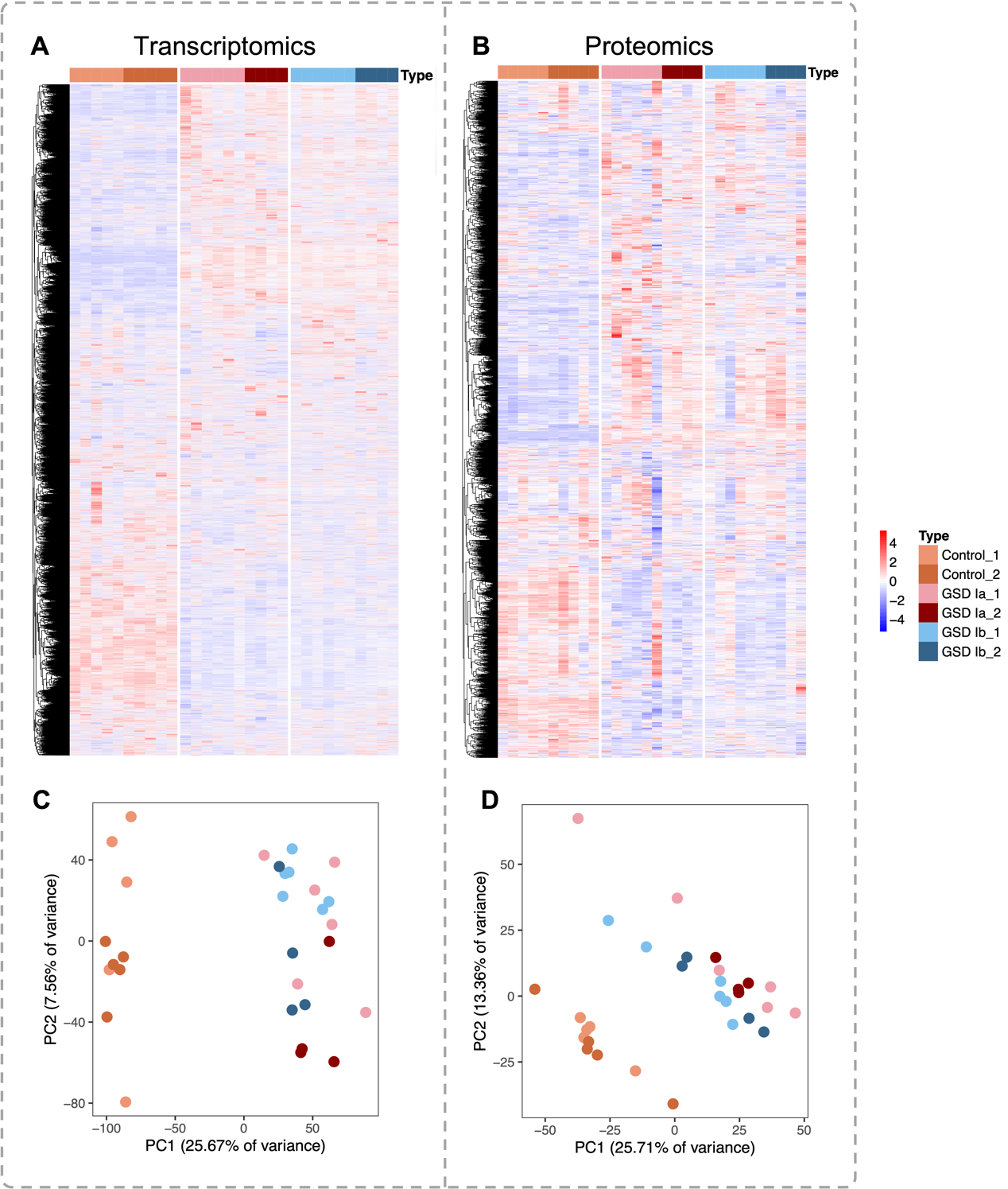
Figure S2.** Heatmaps showing z-score intensity of all the (A) genes and (B) proteins detected.

**
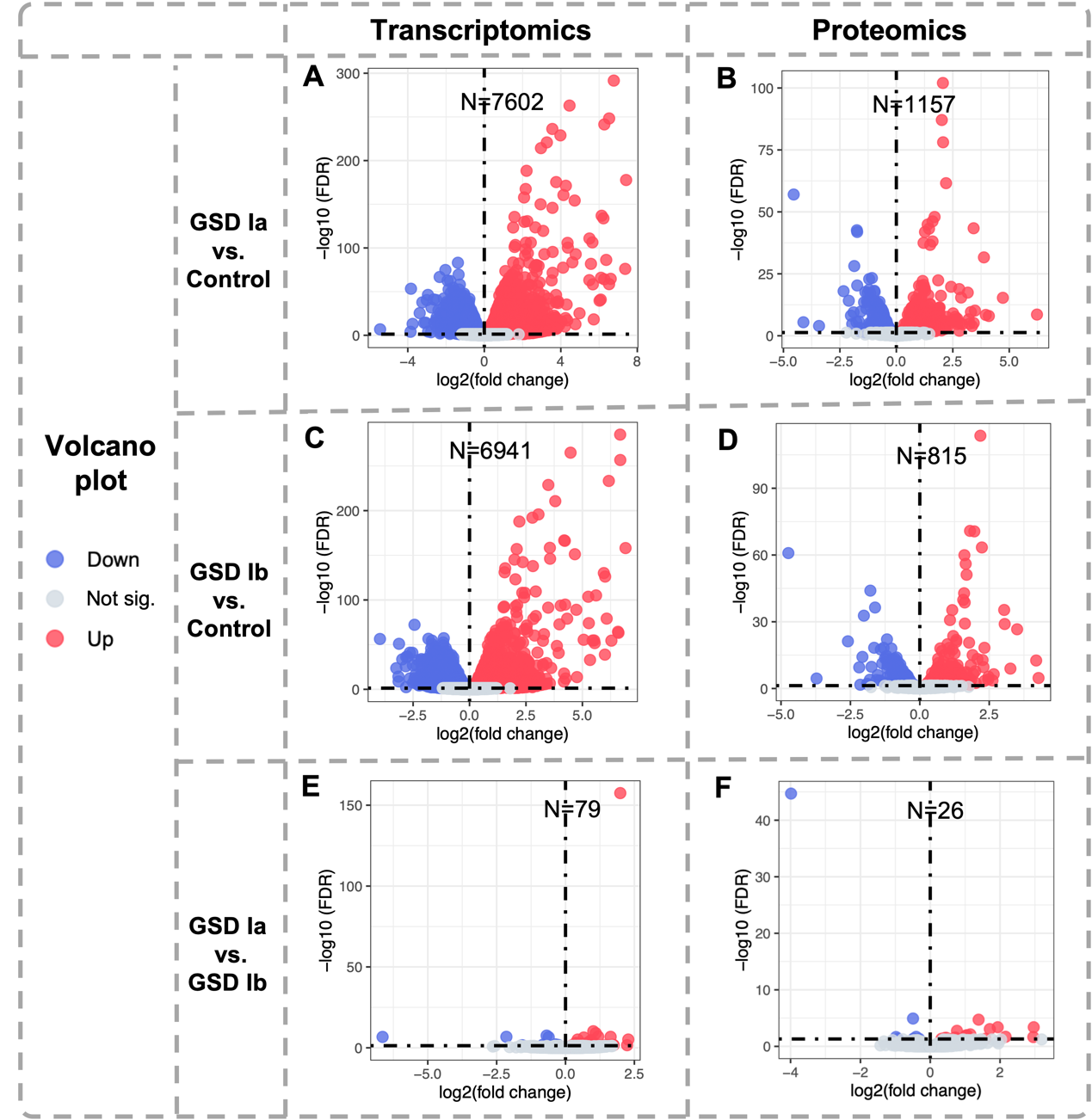
F****igure S3.** Volcano plots showing differential gene and protein groups between GSD Ia versus control, GSD Ib versus control, and GSD Ia versus GSD Ib. ‘N’ represents the number of significantly changed genes or proteins. ‘Not sig.’ refers to proteins that show no significant changes.
